# Synovial Chondromatosis of the Knee Joint: Management With Arthroscopy-Assisted "Sac of Pebbles" Extraction and Synovectomy

**DOI:** 10.7759/cureus.69378

**Published:** 2024-09-13

**Authors:** Rajabalaji Vasudevan, Harishbabu Jayakaran, Munis Ashraf, Navin Balasubramanian

**Affiliations:** 1 Orthopedics, Saveetha Medical College and Hospital, Saveetha Institute of Medical and Technical Sciences, Chennai, IND

**Keywords:** arthroscopy, histopathological examination, loose bodies, osteoarthritis, range of movements, synovial chondromatosis, synovial metaplasia

## Abstract

Synovial chondromatosis is a rare, benign condition characterized by the formation of intra-articular cartilaginous loose bodies, which arise as a result of synovial membrane metaplasia. Due to pain, edema, and joint dysfunction, synovial chondromatosis can result in severe morbidity even though it is a self-limiting condition. In order to avoid problems, such as joint degeneration, early diagnosis and treatment is essential. This case study presents a female patient, age 24, who has experienced increasing pain, swelling, and occasional locking in her left knee for the past two years. Clinical examination of the suprapatellar pouch revealed several palpable loose bodies and effusion. Several intra-articular loose bodies were confirmed by imaging techniques. The patient underwent arthroscopic removal of multiple loose bodies and synovectomy. Histopathological examination confirmed the diagnosis as primary synovial chondromatosis. Postoperatively, the patient achieved a full range of movement without pain, and recurrence was not observed after three months of follow-up. For the treatment of knee synovial chondromatosis, an efficient and less invasive method is the arthroscopy-assisted removal of loose bodies and synovectomy. The value of this method in treating this uncommon condition is emphasized by the significant functional recovery and the lowered risk of recurrence.

## Introduction

Synovial chondromatosis is a rare, benign condition characterized by the metaplastic transformation of the synovial membrane, resulting in the formation of multiple cartilaginous bodies intra-articularly. This condition predominantly affects males, particularly between the ages of 20 and 50, with an incidence of about one in 100,000 population. Although considered non-aggressive and self-limiting, synovial chondromatosis can lead to significant joint damage if not properly managed. The knee joint is most commonly involved, though the hip, elbow, shoulder, and ankle can also be affected [1]. Clinically, the disease presents with a gradual onset of joint stiffness and localized discomfort, typically affecting only one joint.

As the condition progresses, these symptoms can worsen, leading to more severe complications such as joint locking, limited range of movements, joint effusions, and crepitus during movement. In some cases, untreated synovial chondromatosis may lead to the development of secondary osteoarthritis. The underlying cause of this condition remains unclear, but it is believed to involve abnormal synovial tissue proliferation [2]. These nodules get detached from the synovium and become loose bodies, causing mechanical obstruction in the joint.

Early diagnosis and prompt intervention are crucial in managing synovial chondromatosis. Arthroscopic or open surgical removal of the loose bodies and synovectomy is the preferred treatment. Recurrence rates range from 3% to 23% [3]. Malignant chondrosarcoma transformation is an uncommon possibility. Maintaining joint function necessitates early diagnosis and timely intervention.

This case report presents a 24-year-old female patient who was treated with arthroscopic extraction of loose bodies and synovectomy for synovial chondromatosis in her knee joint.

## Case presentation

**Figure 1:**
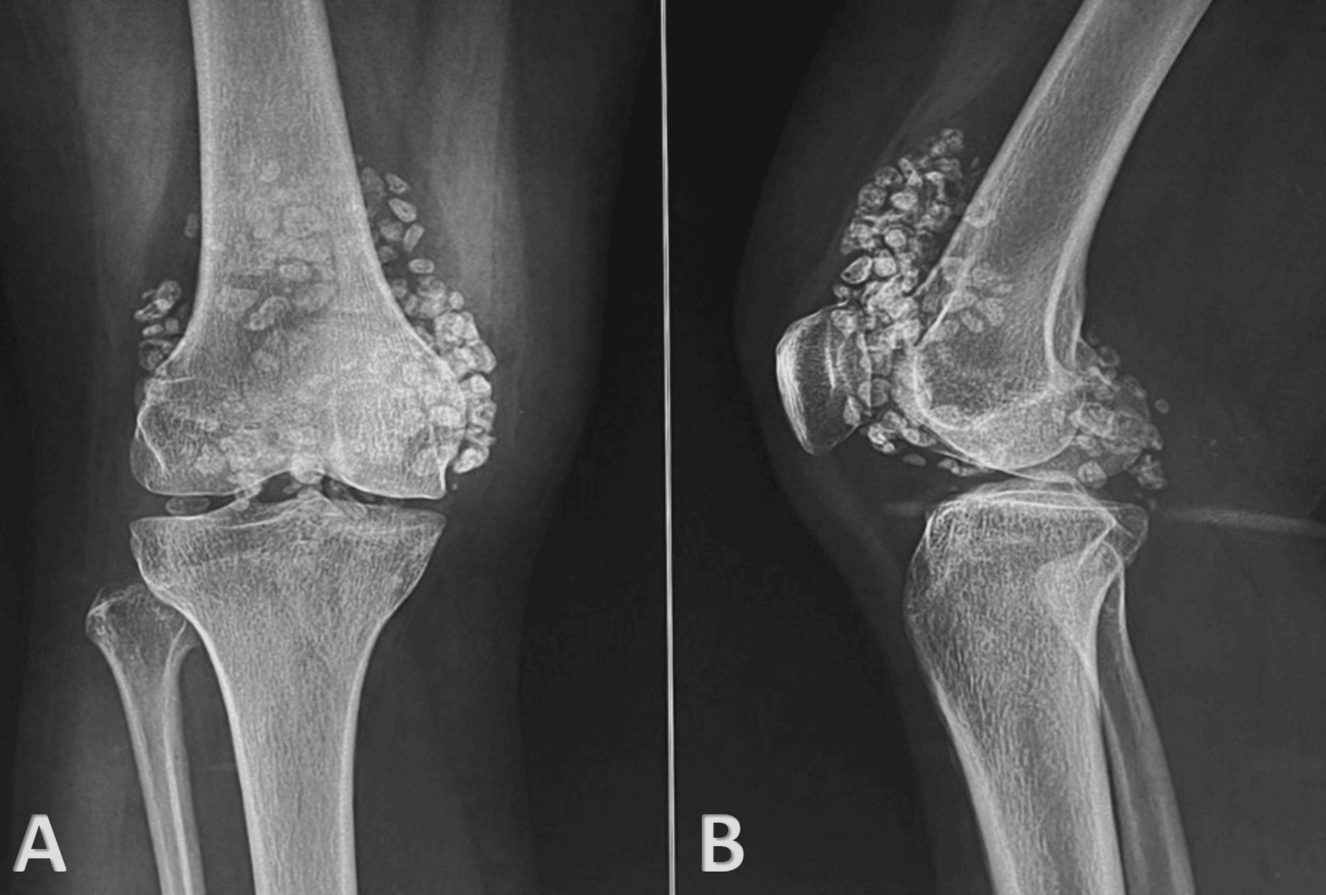
Preoperative radiographs of the left knee joint. (A) Anterior-posterior view of the left knee joint showing multiple loose bodies. (B) Lateral view of the left knee joint showing multiple loose bodies.

**Figure 2:**
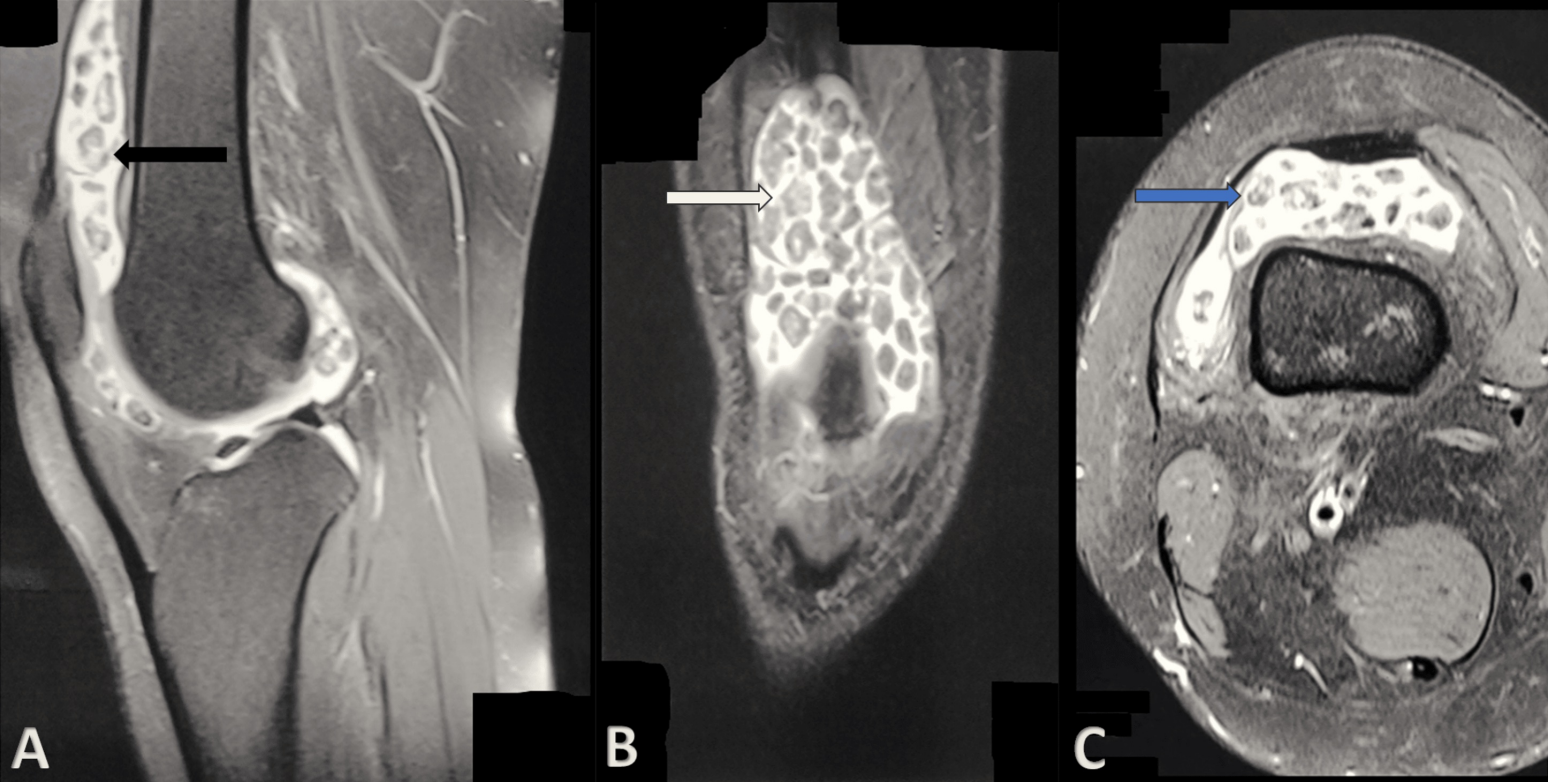
MRI of the left knee joint. (A) MRI sagittal view showing multiple loose bodies in the patellofemoral and tibiofemoral joints (black arrow). (B) MRI coronal view showing multiple loose bodies in the tibiofemoral joint with associated joint effusion (white arrow). (C) MRI axial view showing multiple loose bodies in the patellofemoral joint with associated joint effusion (blue arrow). MRI: magnetic resonance imaging

A 24-year-old woman arrived at the outpatient department, complaining of two years of left knee pain, locking, and swelling. Early on, the symptoms were mild, but they grew worse with time. The patient had no prior history of trauma, involvement of other joints, or low back pain. She has no significant family or medical history relevant to her current condition. The patient has no known comorbidities and no history of allergies. Ocular, mucocutaneous, gastrointestinal, and urinary problems were not present in the past. There was no history of constitutional symptoms. Erythrocyte sedimentation rate (ESR) and C-reactive protein (CRP) were found negative in the patient's medical records. Negative results were obtained for rheumatoid factor (RF), anti-cyclic citrullinated peptide antibody (anti-CCP antibody), anti-nuclear antibody (ANA), and human leukocyte antigen-B27. Analysis of synovial fluid revealed no signs of tuberculosis (TB) and was normal. After the patient's limb was examined, it was found that the swelling was diffuse and non-tender, with evidence of effusion over the suprapatellar pouch and no localized rise in temperature. The patient's limb was held in a 10-degree flexion. The suprapatellar pouch has multiple easily moved and perceptible hard loose bodies. Left knee plain radiograph and magnetic resonance imaging (MRI) were done, which showed calcific loose bodies intra-articularly and anterior to the superior aspect, with a small number in the posterior aspect of the knee (Figure 1 and Figure 2). 

**Figure 3:**
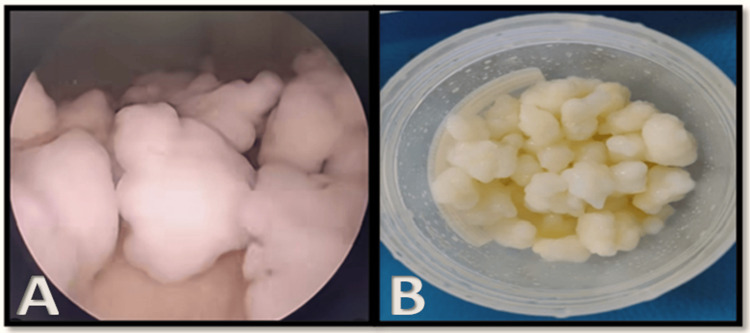
(A) Intraoperative arthroscopic image showing multiple loose bodies in the knee joint. (B) Postoperative image of extracted loose bodies.

It was planned to do surgical management to remove the mechanical block. Arthroscopic-assisted extraction of loose bodies with synovectomy was done, and both the synovial tissue and loose bodies were sent for histopathological analysis (Figure 3).

**Figure 4:**
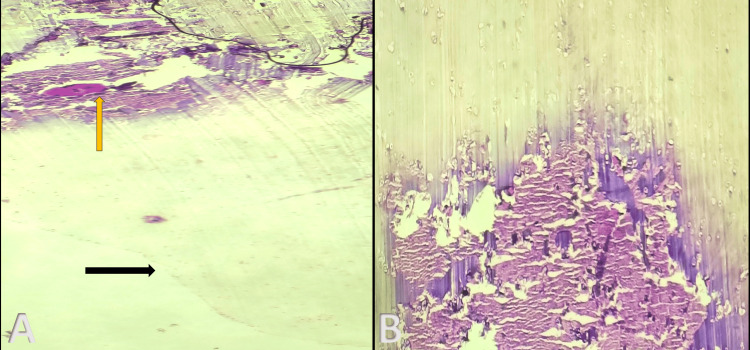
Histopathology images. (A) The black arrow indicates the fragment of the thickened synovial tissue, while the yellow arrow indicates the synovial tissue with foci of calcification. (B) Histopathology image shows the cartilage in the periphery with the central area of calcification.

Under a microscope, synovial tissue containing mature hyaline cartilage islands was seen with foci of ossification. There was no indication of malignancy. The diagnosis of synovial chondromatosis was confirmed by histopathology (Figure 4).

**Figure 5:**
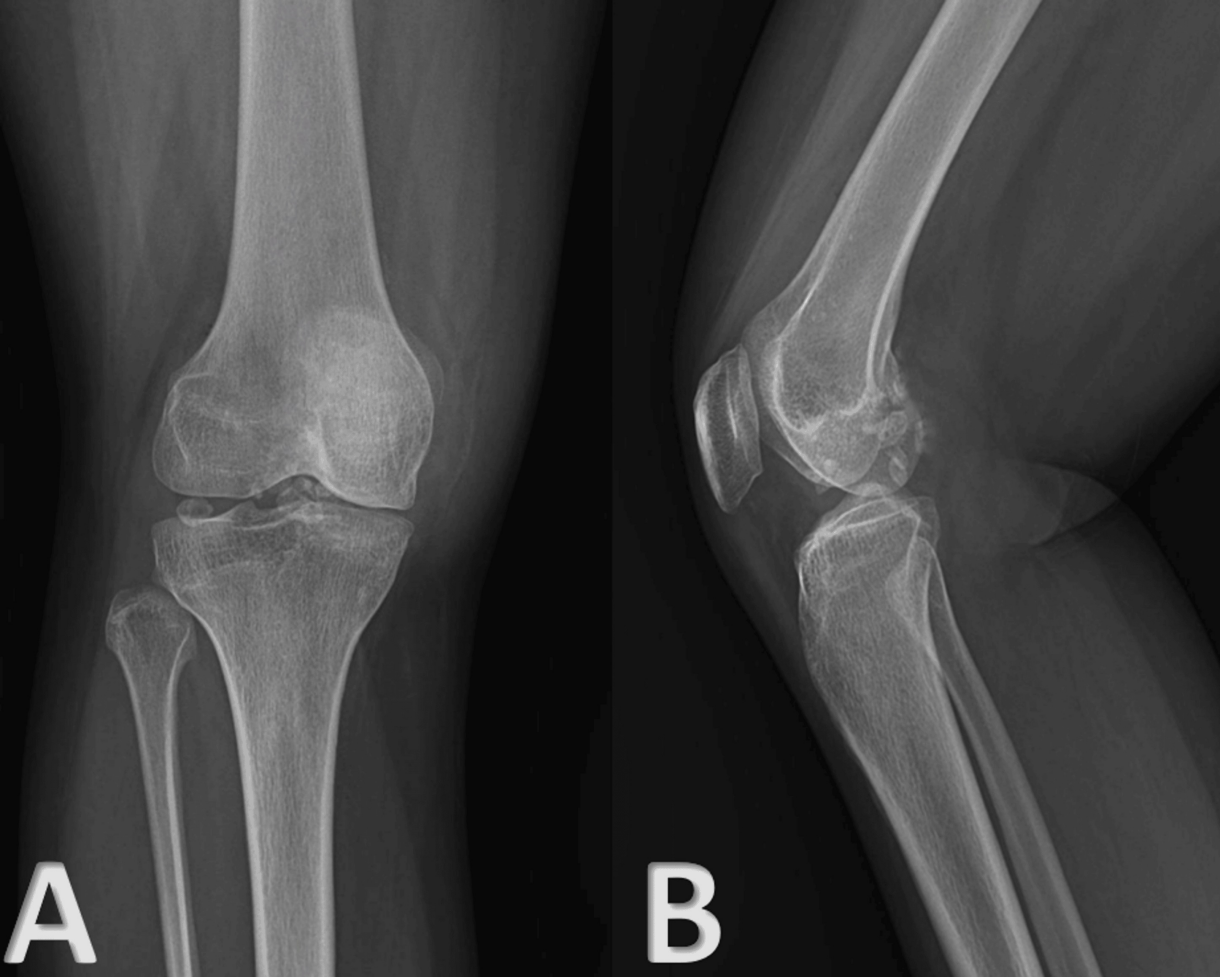
Postoperative radiographs of the left knee joint. (A) Anterior-posterior view of left knee joint. (B) Lateral view of left knee joint.

Following surgery, the patient received instructions on knee mobilization and strengthening exercises, as well as passive and active physical therapy activities. Three months following surgery, the patient's range of movements was 0-135° in flexion without experiencing any pain, and there was no recurrence (Figure 5).

## Discussion

A rare and benign condition known as synovial chondromatosis shows the development of cartilaginous nodules and synovial membrane in bursae, tendons, sheaths, and joints. Although the exact cause of synovial chondromatosis is still unknown, the presence of clonal chromosomal abnormalities in affected tissues suggests a neoplastic origin rather than a purely reactive condition. Based on chromosomal evidence, some researchers have proposed that synovial chondromatosis may represent a benign tumor, where synovial metaplasia leads to the transformation of tissue into cartilage-forming cells. This metaplasia may ultimately lead to the development of cartilaginous bodies intra-articularly, some of which may eventually ossify.

Milgram's classification of synovial chondromatosis is a widely recognized system for analyzing the progression of the disease, based on both pathological and radiographic observations. In the early stage, the synovium is actively diseased, but there are no free-floating loose bodies in the joint. Patients may experience joint pain, swelling, and stiffness, but these symptoms are often nonspecific and can be mistaken for other joint pathologies. As the disease progresses to the transitional stage, the synovium continues to produce cartilaginous nodules, and some of these may separate and form loose bodies within the joint space. During this stage, patients may experience more pronounced symptoms, including mechanical issues such as joint locking or catching due to loose bodies. This stage is critical for diagnosis, as the presence of loose bodies in imaging studies can help differentiate synovial chondromatosis from other joint disorders. In the late stage, the synovium may no longer actively produce new cartilaginous nodules, yet loose bodies remain within the joint. These loose bodies can continue to cause mechanical symptoms and may contribute to secondary degenerative changes in the joint, including osteoarthritis. At this stage, the disease might be mistaken for other conditions associated with loose bodies in the joint, such as osteochondritis dissecans or traumatic osteochondral fractures.

The diagnosis and treatment of synovial chondromatosis depend mainly on imaging, which offers a variety of modalities for determining the disease's severity. Radiopaque loose bodies can be found using plain radiographs, but they work best later in the disease when the bodies have hardened. Non-ossified cartilaginous nodules may not show up on X-rays in the early stages. When evaluating joint effusion or suspected synovial pathology, ultrasound is a helpful non-invasive method that can be used to detect loose bodies and evaluate synovial thickness. When deciding between arthroscopic and open surgical techniques, computed tomography (CT) imaging is very useful for surgical planning because it offers extensive information about the size, number, and location of calcified loose bodies. Due to its excellent soft tissue contrast, MRI is the preferred imaging method for diagnosing synovial chondromatosis. A thorough assessment of the synovium, the identification of calcified and noncalcified nodules, and the evaluation of related joint pathology, such as effusion or cartilage loss, are all made possible by MRI. Furthermore, MRI helps in early diagnosis and efficient treatment planning by detecting disease in its early stages before loose bodies have calcified.

The primary treatment for synovial chondromatosis is surgery, aiming to remove the mechanical block by loose bodies and to address the diseased synovium to prevent recurrence. Surgical options include arthroscopic extraction of loose bodies with synovectomy, which is a minimally invasive procedure that facilitates synovectomy and the removal of loose bodies under direct visualization [4,5]. When the disease is limited to one or a few compartments and the loose bodies are accessible, arthroscopy is frequently chosen for early and transitional stages. By removing the affected synovium, an arthroscopic synovectomy lowers the chance of a recurrence [6,7]. If the disease is widespread or affects multiple compartments that are difficult to access arthroscopically, an open synovectomy can be required [8]. Though more intrusive and requiring a longer recovery period, this method allows for the full removal of the afflicted synovium and extraction of any loose bodies. Total knee arthroplasty can be an option for patients whose condition is advanced or if their synovial chondromatosis is associated with severe osteoarthritis [9]. Joint function is enhanced and pain is reduced with total knee arthroplasty that treats both the synovial chondromatosis and the underlying degenerative abnormalities. However, older patients or those with serious joint injuries are usually the ones who get total knee arthroplasty [10].

When detected early and treated properly, synovial chondromatosis is generally regarded as a non-aggressive disorder with a fair prognosis. There is a chance of a local recurrence, nevertheless, especially if the synovium is not completely removed during the procedure. The importance of histopathological examination of excised tissue is underscored by the rare instances of malignant transformation from synovial chondromatosis to synovial chondrosarcoma. Adjunctive treatments, such as chemotherapy and radiotherapy, have been investigated as a means of preventing recurrence in instances that are aggressive or recurring; however, their effectiveness is still being studied [11].

Given the nonspecific symptoms and radiologic findings associated with synovial chondromatosis, it is important to consider other conditions in the differential diagnosis. Trauma-related conditions such as fractures with avulsed fragments can produce loose bone fragments that resemble the loose bodies seen in synovial chondromatosis. This can be distinguished by a history of trauma and the presence of fracture lines on imaging. Similarly, fragmentation of the meniscus with calcification can result in loose bodies within the joint, though these are typically associated with meniscal tears and degenerative changes. Degenerative joint disease, particularly with detached osteophyte spurs, can also produce loose bodies, especially in advanced osteoarthritis. Imaging findings such as subchondral sclerosis and joint space narrowing help support the diagnosis of degenerative joint disease. Synovial proliferation disorders like pigmented villonodular synovitis (PVNS) should also be considered, as PVNS is a benign proliferative synovial disease that can cause joint pain and swelling [12]. PVNS often exhibits hemosiderin deposition, visible on MRI as low-signal intensity areas, which helps differentiate it from synovial chondromatosis. Finally, neoplastic conditions like synovial chondrosarcoma, though rare, must be considered. Synovial chondrosarcoma is a malignant tumor that may initially resemble synovial chondromatosis but is distinguished by its aggressive behavior, rapid growth, bone destruction, and soft tissue invasion. Differentiating benign synovial chondromatosis from malignant synovial chondrosarcoma requires a thorough histopathological examination.

## Conclusions

Synovial chondromatosis is a rare condition. Due to its nonspecific clinical symptoms, synovial chondromatosis in the knee is difficult to diagnose and is typically delayed, especially before ossifying nodules develop. Secondary degenerative osteoarthritis of the knee can result from loose bodies within the joint. Here, we describe a case of knee synovial chondromatosis that was managed using an arthroscopy-assisted synovectomy and loose body extraction. Arthroscopy, in our opinion, is a simple and effective way to treat this condition.
